# Complete Genome Sequence of *Mycobacterium avium*, Isolated from Commercial Domestic Pekin Ducks (*Anas platyrhynchos domestica*), Determined Using PacBio Single-Molecule Real-Time Technology

**DOI:** 10.1128/genomeA.00769-16

**Published:** 2016-09-01

**Authors:** Xiao-Heng Song, Hong-Xi Chen, Wang-Shu Zhou, Jiang-Bo Wang, Ma-Feng Liu, Ming-Shu Wang, An-Chun Cheng, Ren-Yong Jia, Shun Chen, Kun-Feng Sun, Qiao Yang, Ying Wu, Xiao-Yue Chen, De-Kang Zhu

**Affiliations:** a Research Center of Avian Diseases, College of Veterinary Medicine of Sichuan Agricultural University, Chengdu, Sichuan, China; b Key Laboratory of Animal Disease and Human Health of Sichuan Province, Chengdu, Sichuan, China; c Institute of Preventive Veterinary Medicine of Sichuan Agricultural University, Chengdu, Sichuan, China

## Abstract

*Mycobacterium avium* is an important pathogenic bacterium in birds and has never, to our knowledge, reported to be isolated from domestic ducks. We present here the complete genome sequence of a virulent strain of *Mycobacterium avium*, isolated from domestic Pekin ducks for the first time, which was determined by PacBio single-molecule real-time technology.

## GENOME ANNOUNCEMENT

*Mycobacterium avium* infection is a contagious disease of various species of birds, including domestic poultry, pet and exotic birds, and free-living and captive wild birds (1). However, no *Mycobacterium avium* strain has been isolated from domestic birds in the United States (2). *Mycobacterium avium* induces progressive emaciation, decreased egg production, and finally causes death in birds (2). Avian tuberculosis is reported to be extremely rare in commercial duck flocks, which may be related to ducks that are moderately resistant to *Mycobacterium avium* (3). We first reported a case of avian tuberculosis outbreaks in commercial domestic ducks (4), and we isolated a strain of *Mycobacterium avium*. To gain insights into its virulence and resistance, we sequenced the complete genome of the isolated strain.

Genomic DNA of *Mycobacterium avium* was extracted (5) and sequenced using single-molecule real-time sequencing with PacBio RS II sequencer (6). The filtered subread data were used in the assembly process MHAP to obtain a *de novo* assembly (7, 8). The assembled sequence was annotated by the NCBI Prokaryotic Genome Annotation Pipeline (PGAP). Clustered regularly interspaced short palindromic repeats (CRISPRs) were predicted by the PILER-CR software and CRISPRFinder (9, 10). Secretory protein prediction was done using the KohGPI software. Resistance gene identification was performed using the Comprehensive Antibiotic Resistance Database (CARD) (11), and virulence gene identification was done using the Virulence Factor Database (VFDB) (12). The results showed the total length of genome to be 4,565,181 bp. The genome sequence has a mean G+C content of 69.3%, the sequencing depth was 15×, and there were 4,616 protein-coding regions, 33 tRNA genes, 70 rRNA genes, and three micro RNA (miRNA) genes, but no CRISPR arrays.

Preliminary analysis of the annotated genome finds several vital virulence factors: the Mce family protein, which is related to invasion and survival in host cells; and BrkB and mvin family proteins, which are related to membrane protein synthesis, in which there are 154 secretory proteins. In addition, there is quinolone resistance protein NorA, multidrug resistance protein EmrB, multidrug resistance protein MarR, and tetracenomycin C resistance and export protein TcmA, which belong to the major facilitator superfamily (MFS). β-Lactamase CcrA, penicillin-binding protein (PBP), and metallo-β-lactamase, conferring resistance to β-lactamase antibiotics, are also found. A novel mutation was observed in the *embC* and *embB* genes coding for ethambutol resistance. These suggest that this strain is extensively drug resistant. Besides, this strain has transposase A, transposase B, and transposase mutator family proteins, which may be an important reason for many resistance genes in it. This is the first report of whole-genome sequencing of *Mycobacterium avium* isolated from domestic Pekin ducks (*Anas platyrhynchos domestica*).

### Accession number(s).

This complete genome project has been deposited in DDBJ/ENA/GenBank under the accession no. CP016396. The version described in this paper is the first version. The complete genome raw sequence reads were deposited in the Sequence Read Archive under the accession no. SRR3501121.
